# P-1551. Prevalence and Clinical Outcomes of RSV in Adults with Severe Acute Respiratory Infection (SARI)

**DOI:** 10.1093/ofid/ofaf695.1731

**Published:** 2026-01-11

**Authors:** Megha Priyadarshi, Sunit Sikdar, Manish Soneja, Megha Brijwal, Aashish Choudhary, Neeraj Nischal, Animesh Ray, Lalit Dar, Naveet Wig, Maroof Ahmad Khan

**Affiliations:** All india institute of medical sciences, Delhi, Delhi, India; All India Institute of Medical Sciences, New Delhi, Delhi, Delhi, India; All India Institute of Medical Sciences, New Delhi, Delhi, India; All India Institute of Medical Sciences, New Delhi, India, New Delhi, Delhi, India; All India Institute of Medical Sciences, New Delhi, Delhi, Delhi, India; All India Institute of Medical Sciences, New Delhi, Delhi, Delhi, India; All India Institute of Medical Sciences, New Delhi, Delhi, Delhi, India; All India Institute of Medical Sciences, New Delhi, India, New Delhi, Delhi, India; All India Institute of Medical Sciences, New Delhi, Delhi, India; All India Institute of Medical Sciences, New Delhi, Delhi, Delhi, India

## Abstract

**Background:**

Respiratory syncytial virus (RSV) is an increasingly recognized cause of severe acute respiratory infection (SARI) among adults. Despite its clinical significance, data on RSV prevalence and outcomes in adults from India remain limited. With recent FDA approval of RSV vaccines for older adults, understanding regional epidemiology is critical for guiding vaccination strategies and clinical management.

**Figure:**
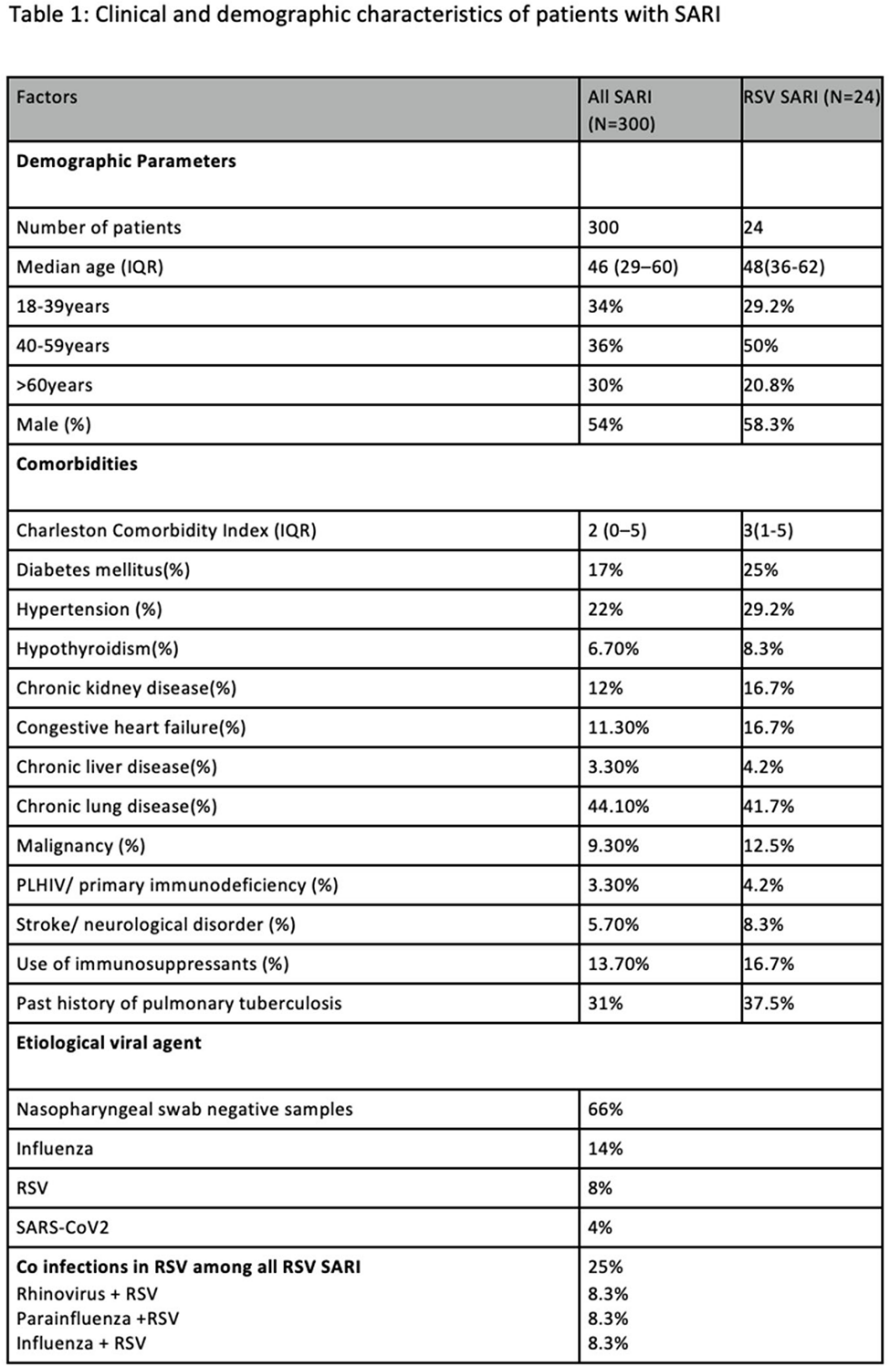

**Figure:**
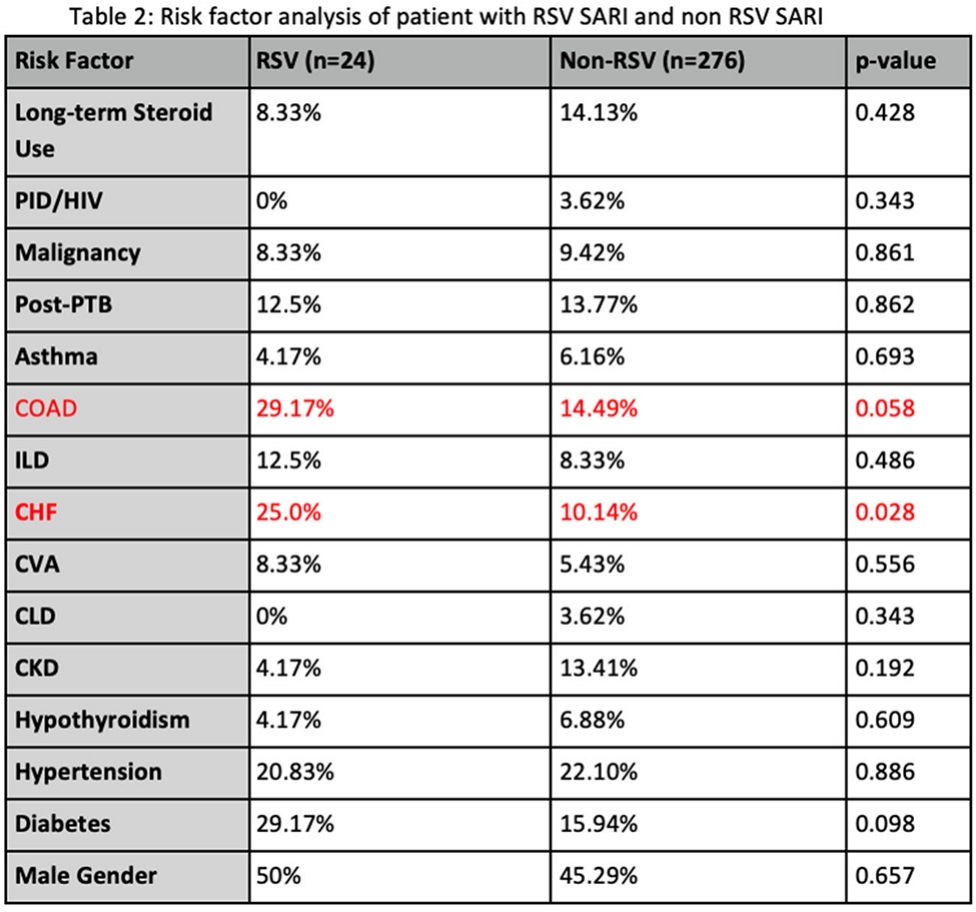

**Methods:**

A prospective observational study was conducted at AIIMS, New Delhi, from November 2023 to January 2025. Adults aged ≥18 years hospitalized with SARI were enrolled consecutively. Clinical, demographic, and laboratory data were collected using a structured proforma. Respiratory specimens (nasal/throat swabs and lower respiratory tract samples) were tested by real-time RT-PCR for RSV, influenza, and other respiratory viruses. RSV-positive patients were followed during hospitalization to assess clinical outcomes, including oxygen requirement, complications, and mortality.

**Figure:**
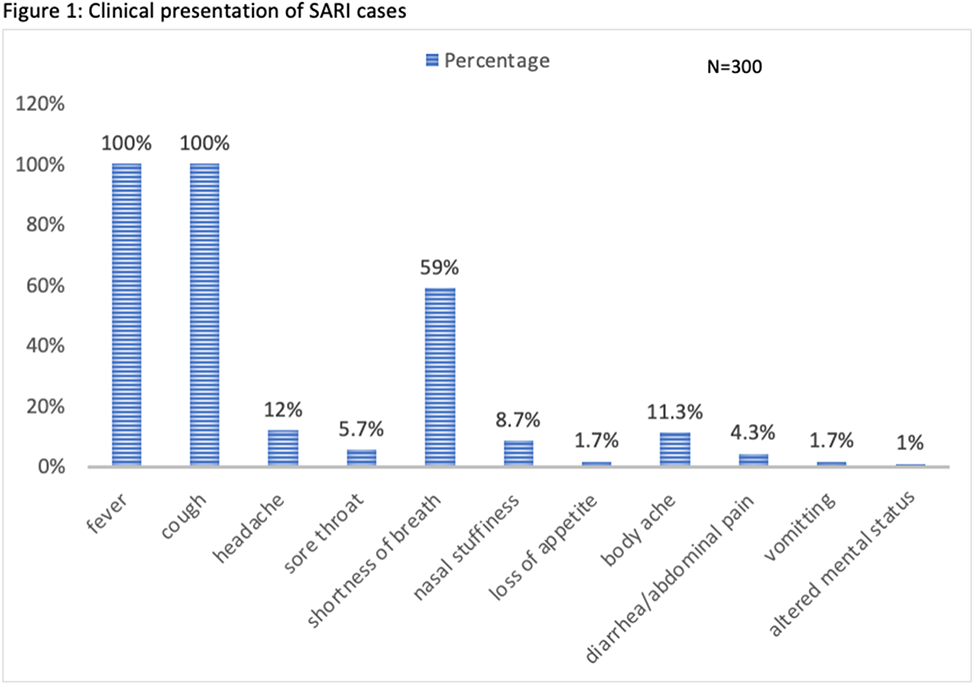

**Figure:**
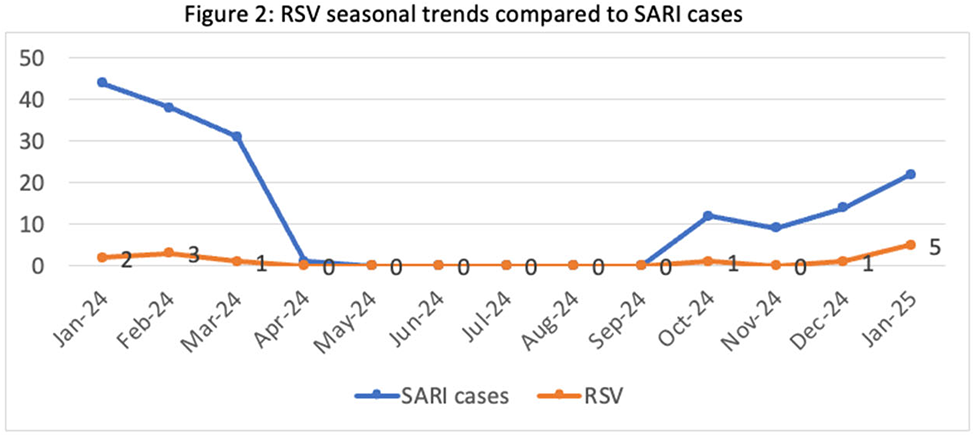

**Results:**

Among 300 SARI patients (median age 46 years; 54% male), RSV was identified in 8% (all serotype B). Common comorbidities were chronic lung disease (44.1%), hypertension (22%), and diabetes (17%). Compared to non-RSV cases, RSV-positive patients more frequently had CHF (25% vs 10.1%), COAD (29.2% vs 14.5%), and diabetes (29.2% vs 15.9%). No RSV patients had HIV or CLD. Viral Coinfections were seen in 25% of RSV SARI patients and secondary bacterial infection also in 25% patients, with carbapenem-resistant Acinetobacter baumannii predominant. Overall mortality was 19.3%, slightly higher in RSV-positive cases (20.8%). No significant differences were noted in ICU admission, mechanical ventilation, or mortality between RSV and non-RSV SARI patients.

**Conclusion:**

RSV contributes significantly to the burden of SARI in Indian adults. Early identification of RSV infection, preventive strategies such as vaccination for high-risk populations, and targeted clinical management could potentially reduce the morbidity and mortality associated with RSV.

**Disclosures:**

All Authors: No reported disclosures

